# The gut homeostasis-immune system axis: novel insights into rheumatoid arthritis pathogenesis and treatment

**DOI:** 10.3389/fimmu.2024.1482214

**Published:** 2024-09-26

**Authors:** Peng Qi, Xin Chen, Jiexiang Tian, Kexin Zhong, Zhonghua Qi, Menghan Li, Xingwen Xie

**Affiliations:** ^1^ Gansu University of Traditional Chinese Medicine, Lanzhou, China; ^2^ Affiliated Hospital of Gansu University of Traditional Chinese Medicine, Lanzhou, China

**Keywords:** gut homeostasis, immune system, rheumatoid arthritis, pathogenesis, novel therapeutic approaches

## Abstract

Rheumatoid arthritis is a widely prevalent autoimmune bone disease that imposes a significant burden on global healthcare systems due to its increasing incidence. In recent years, attention has focused on the interaction between gut homeostasis and the immune system, particularly in relation to bone health. Dysbiosis, which refers to an imbalance in the composition and function of the gut microbiota, has been shown to drive immune dysregulation through mechanisms such as the release of pro-inflammatory metabolites, increased gut permeability, and impaired regulatory T cell function. These factors collectively contribute to immune system imbalance, promoting the onset and progression of Rheumatoid arthritis. Dysbiosis induces both local and systemic inflammatory responses, activating key pro-inflammatory cytokines such as tumor necrosis factor-alpha, Interleukin-6, and Interleukin-17, which exacerbate joint inflammation and damage. Investigating the complex interactions between gut homeostasis and immune regulation in the context of Rheumatoid arthritis pathogenesis holds promise for identifying new therapeutic targets, revealing novel mechanisms of disease progression, and offering innovative strategies for clinical treatment.

## 1 Introduction

Rheumatoid arthritis (RA) is a multifaceted, chronic autoimmune disorder characterized by persistent inflammation across multiple joints, leading to joint damage and functional impairment. This condition is typified by the production of autoantibodies, chronic synovitis, and sustained inflammation (1). Affecting approximately 1% of the global population, RA exhibits a higher prevalence among females (2). Hallmark manifestations include widespread joint swelling, tenderness, and systemic inflammation driven by autoantibodies (2). The inflammatory pathways in RA are marked by alterations in the T helper 1 cell profile, which result in an imbalance between anti-inflammatory and pro-inflammatory cytokines (2). Autoantibodies linked to RA, such as rheumatoid factor, anti-citrullinated protein antibodies, and anti-carbamylated antibodies, can be detected in serum long before the clinical manifestation of the disease (3, 4). Although joint involvement is a hallmark of RA. Affected joint spaces exhibit the release of cytokines such as Interleukin-1, Interleukin-6, and tumor necrosis factor-alpha, accompanied by reduced levels of Interleukin-11,Interleukin-13, and Interleukin-10 (IL-10) (5). However, the pathogenesis of RA may involve extra-articular sites, including the lungs, periodontal tissues, gut microbiota, or citrullination processes in adipose tissue. This may also explain why some anti-citrullinated protein antibody-positive patients with joint pain present with normal synovial tissue (6).

In recent years, microbial factors have garnered significant attention for their association with the pathogenesis of RA. Patients with RA exhibit notable dysbiosis of the oral microbiome, which can be partially restored through RA treatment, with the extent of recovery closely correlated with the patient’s therapeutic response (7). Epidemiological studies reveal that periodontitis, a chronic infectious oral disease, is highly prevalent among RA patients and is strongly linked to the disease. *Porphyromonas gingivalis*, a key pathogen in periodontitis, has been implicated in the development of RA due to its ability to synthesize bacterial peptidylarginine deiminase (7). This enzyme induces citrullination of α- and β-fibrin chains within the synovium, generating autoantigens that promote the production of anti-citrullinated protein antibodies (7). These anti-citrullinated protein antibodies form immune complexes with citrullinated proteins, which bind to fragment crystallizable region and complement component 5a receptors on immune and inflammatory cells, triggering a complex cascade of immune responses and the release of inflammatory mediators, ultimately resulting in synovial inflammation and the onset of RA (8).

The gut microbiota plays a pivotal role in modulating host immune responses and is intricately linked to the development of several autoimmune conditions (2). The complex interplay between gut health and immune system homeostasis may be a crucial determinant in the etiology and progression of RA. Regulation of gut and immune system homeostasis is vital for overall health. Gut homeostasis involves the composition, function, and stability of the gut microbiome, as well as the integrity of both physical and immune barriers (9). The gut microbiota, often considered an invisible organ, plays a crucial role in maintaining intestinal barrier function, nutrient absorption, and overall immune and metabolic balance (10, 11). One of its primary metabolic functions is aiding the digestion of indigestible food residues, such as polysaccharides, oligosaccharides, and unabsorbed sugars and alcohols (12). The intestinal barrier consists of multiple layers: an outer mucus layer, a symbiotic gut microbiota, defense proteins like antimicrobial peptides and secretory immunoglobulin A; a middle layer of intestinal epithelial cells; and an inner layer of innate and adaptive immune cells (13, 14). The mucosal immune system helps protect the body by activating and regulating effector cells. When this immune system is disrupted, pathogenic flora can escape the intestinal tract (15). Evidence suggests that gut microbiome dysbiosis is a critical environmental factor in RA pathogenesis and may provoke abnormal immune responses (16, 17). Gut microbiota dysbiosis increases intestinal mucosal permeability, elevating endotoxin levels in the bloodstream and disrupting the balance of downstream metabolites that regulate systemic inflammation. These disruptions can initiate chronic systemic inflammatory responses, potentially influencing the onset and progression of osteoarthritis (18). Although the role of the gut-immune homeostatic axis in RA has been explored, its intricate regulatory network is not yet fully understood. Further research into how the gut microbiota and its metabolites affect RA is valuable for understanding RA pathogenesis and developing novel treatment strategies.

## 2 Interactions between gut homeostasis and the immune system

**Figure 1:**
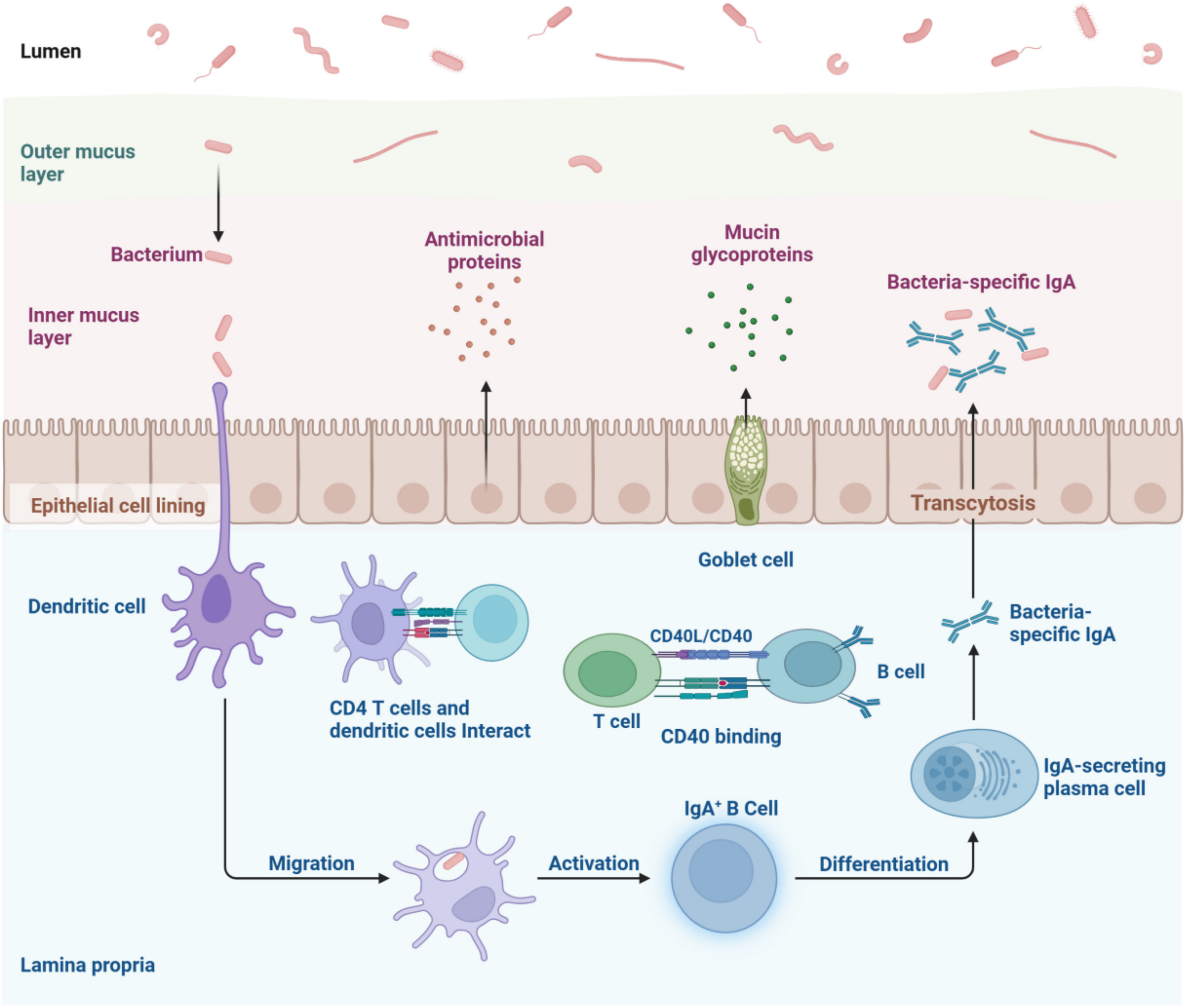
The intestinal mucosal immune network represents a intricate system comprising innate, adaptive, and immunoglobulin A-mediated immune mechanisms. Epithelial cells and dendritic cells recognize antigens via pattern recognition receptors and present them to T and B lymphocytes within lymphoid tissues, triggering adaptive immune responses. Simultaneously, they activate signaling cascades, stimulate the production of cytokines and antimicrobial peptides, and mount acute inflammatory reactions to eliminate offending agents. B cells differentiate into plasma cells that secrete immunoglobulin A, which is then transported into the gut lumen, where it neutralizes antigens and pathogens through its binding capabilities.

Gut microbes are essential for sustaining immune system balance. Changes in the gut microbiota and their metabolites can trigger inflammatory disorders related to the immune system, while a compromised intestinal barrier increases permeability and stimulates immune responses (19). The gastrointestinal tract houses the majority of the body’s immune cells and constantly interacts with the gut microbiota, influencing their function and characteristics. The gut microbiota’s delicate equilibrium in fostering either commensal or symbiotic relationships enables continuous bidirectional communication with the host immune system (20). System is shown in **Figure 1**.

### 2.1 Interactions between the gut microbiome and the immune system

The interactions between gut microbes and the host immune system are complex and context-dependent. The critical role of the gut microbiome in immune system development and response has been well established (21, 22). The microbiota is essential for regulating immune cell activities and inflammatory cytokines, thereby normalizing immune responses (23). Various gut microbes modulate immune responses differently. For example, segmented filamentous bacteria (SFB) promote the development of intestinal helper T cells 17 (Th17), and the SFB-dependent Th17 response inhibits bacterial translocation in mice with constitutively activated myosin light chain kinase (24). SFB enhances autoimmune responses in a context-dependent manner while strengthening intestinal barrier integrity. *Clostridium*, on the other hand, induces regulatory T cells (Treg) (25, 26). Additionally, distinct populations of Th17 cells exist in the gut, with their development largely influenced by different microorganisms. SFB-induced Th17 cells maintain homeostasis, whereas Th17 cells induced by Citrobacter are pro-inflammatory (27). Different bacterial species trigger distinct systemic immune responses. Probiotics like *Lactobacillus* and *Bifidobacterium* promote immune regulation by increasing regulatory T cell numbers, thereby maintaining immune tolerance and suppressing inflammatory responses. In contrast, harmful bacteria, such as those in the *Enterobacteriaceae* family, activate the Nuclear Factor kappa-light-chain-enhancer of Activated B cells signaling pathway and promote the release of pro-inflammatory cytokines, leading to systemic inflammation. Clinical studies have shown that elevated levels of *Prevotella* and *Veillonella* are closely associated with the development of autoimmune diseases. These bacteria exacerbate conditions like rheumatoid arthritis and inflammatory bowel disease by disrupting the gut barrier and facilitating the translocation of pathogens and their metabolites (28). Furthermore, during an inflammatory response, the intestinal microbiota can become disturbed, leading to an increase in harmful bacteria and a decrease in beneficial ones, which may exacerbate inflammation. For instance, the expansion of Pseudomonas aeruginosa causes intestinal inflammation and disrupts epithelial barrier integrity, allowing bacterial antigens to enter systemic circulation and activate immune responses at distant sites (29). Dysbiosis can result in the overactivation of T-helper 1 cells and Th17 cells, leading to excessive production of pro-inflammatory cytokines such as tumor necrosis factor, interleukin-6, and interleukin-17. This triggers systemic inflammation and immune dysregulation. Such abnormal cellular immune responses are pivotal in the pathogenesis and progression of autoimmune diseases like rheumatoid arthritis (19).

### 2.2 Gut microbial metabolite interactions with the immune system

The gut microbiota generates a variety of metabolites, including SCFAs, amines, polyamines, vitamins, and other small molecules. These metabolites are transported through the bloodstream to tissues and organs throughout the body, where they influence immune responses and regulate inflammatory processes. Metabolites derived from gut microbiota are crucial in modulating the development and function of both adaptive and innate immune cells. IL-10 produced by effector T cells is a key self-regulatory mechanism that maintains immune balance (30). Short-chain fatty acids enhance IL-10 production in T-helper 1 Cells cells through a GPR43-dependent pathway and inhibit histone deacetylase histone deacetylase activation during T helper 1 and Th17 differentiation (22). Interleukin-22, a member of the IL-10 family, is vital for preventing intestinal inflammation, with CD4+ T cells being a major source of Interleukin-22 during chronic inflammation. Short-chain fatty acids stimulate Interleukin-22 production in CD4+ T cells via the G protein-coupled receptor 41 pathway and reduce histone deacetylase activity (22). Butyrate-treated dendritic cells support Treg differentiation and inhibit T-helper 1 Cells differentiation by increasing the expression of immunosuppressive enzymes such as indoleamine 2,3-dioxygenase 1 and aldehyde dehydrogenase 1 family member A2, through an SLC5A8-dependent mechanism (31). Microbial tryptophan metabolites, including indole and its derivatives, interact with aryl hydrocarbon receptors and influence B cell development, differentiation, cytokine production, and regulation through aryl hydrocarbon receptors signaling (32–34). Lipopolysaccharide (LPS), a component of Gram-negative bacteria in the gut, These metabolites are able to enter the bloodstream, initiates a cell-factor cascade that contributes to T cell-mediated inflammatory processes (35). Additionally, bile acids and their metabolites modulate immune responses by regulating signaling pathways and balancing Th17 and Treg cells (36).

### 2.3 Interactions between the gut barrier and the immune system

The gut barrier, which includes intestinal epithelial cells, tight junction proteins, and mucus layers (37). The integrity of the gut barrier is vital for preventing pathogen invasion and maintaining immune homeostasis. When this barrier is compromised, undigested food particles, pathogens, and toxins can penetrate into the bloodstream, triggering systemic immune responses and promoting chronic inflammation (38). Dysfunction of the gut barrier has been associated with various immune-mediated diseases, including rheumatoid arthritis and inflammatory bowel disease (39). Gut-resident immune cells, such as dendritic cells, goblet cells, and T cells, are essential for regulating both local and systemic immune responses by detecting and responding to gut microbes and their metabolites (40). Disruption of the gut barrier can cause apoptosis of intestinal epithelial cells and create a pro-inflammatory environment, which includes the activation of autoreactive Th17 cells and other helper T cells (19). Pro-inflammatory cytokines like tumor necrosis factor-alpha and interferon-gamma can damage tight junctions (41–43), whereas immunosuppressive cytokines such as interleukin-10 and transforming growth factor-beta help to preserve their integrity (44). A weakened mucosal immune defense reduces Immunoglobulin A secretion or causes Immunoglobulin A dysfunction, while dysbiosis activates B cells, leading to abnormal levels of pro-inflammatory immunoglobulins like Immunoglobulin G. This can elevate rheumatoid arthritis-specific antibodies, further promoting systemic autoimmune responses (29, 45).

## 3 Impact of the gut homeostasis-immune system axis on rheumatoid arthritis

**Figure 2:**
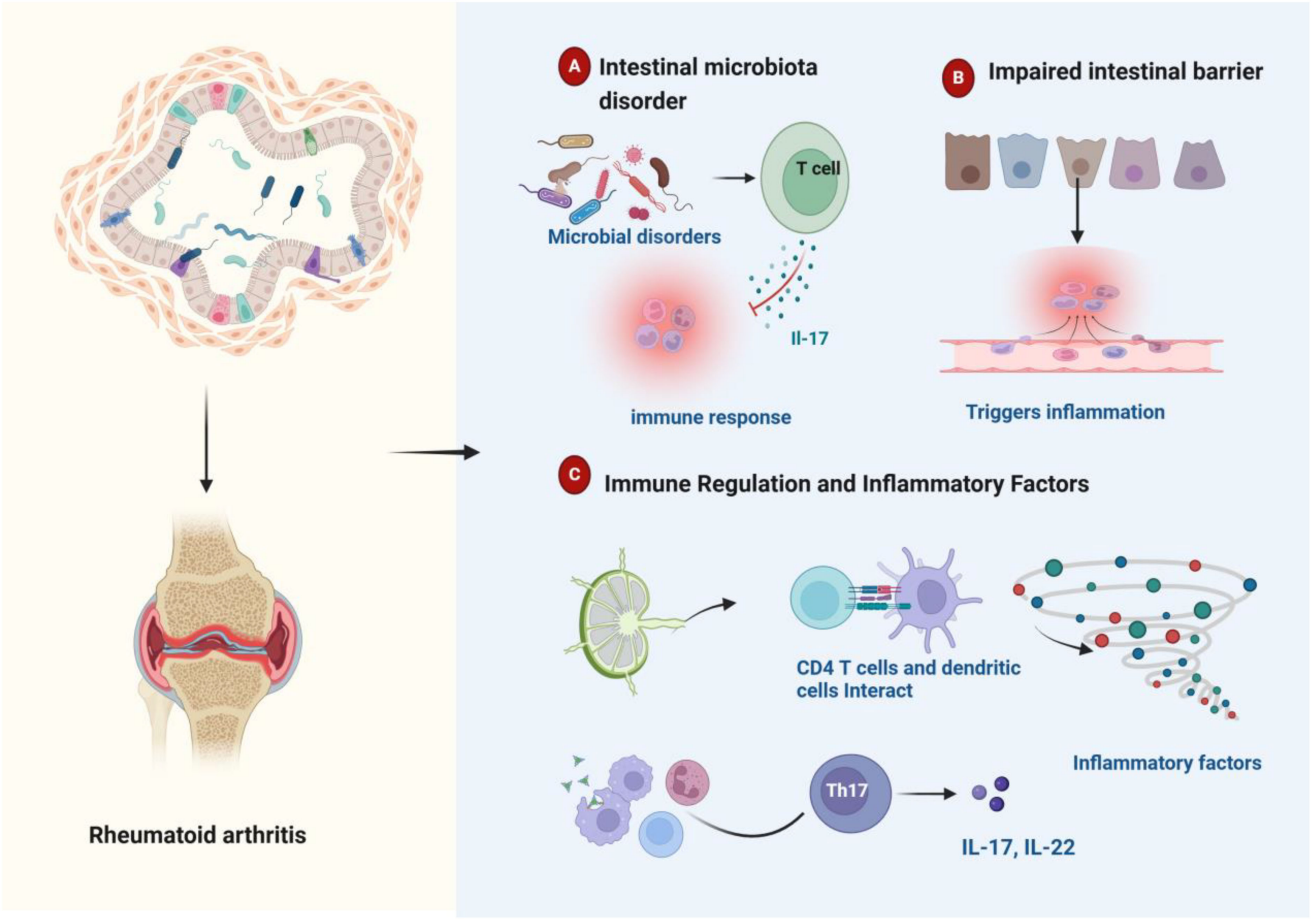
Schematic representation of intestinal homeostasis influencing rheumatoid arthritis pathogenesis. **(A)** Dysbiosis of the gut microbiome can trigger an immune response. **(B)** Damage to the gut barrier can trigger inflammation. **(C)** Immune cells residing in the gut play a regulatory role in modulating inflammatory cytokine production. Gut microbial imbalance is associated with aberrant inflammatory cytokine profiles and dysregulated immune responses, which collectively contribute to the development and progression of rheumatoid arthritis.

RA is complex and multifactorial (46, 47). The human body is home to over 100 trillion microbes, predominantly residing in the gut (48). Advances in bacterial DNA sequencing technology have elucidated the relationship between gut bacteria and RA, underscoring the significant role of the gut microbiota in the disease (29, 47). Research has shown notable differences in the gut microbiome between RA patients and healthy individuals, and these differences can affect RA manifestations (49, 50). The effect of intestinal homeostasis on rheumatoid arthritis is shown in **Figure 2**. Pro-inflammatory gut pathogens reshape the immune landscape by overactivating the innate immune system, which subsequently drives abnormal activation of the adaptive immune system, inducing RA. The gut microbiota composition also modulates sex hormone levels, influencing RA risk. Hormonal deficiencies increase intestinal permeability, elevating Th17 cells, nuclear factor kappa-light-chain-enhancer of activated B cells, Interleukin-17, and tumor necrosis factor-αlevels in peripheral blood, which promotes bone resorption. Gut homeostasis imbalance is closely linked to RA pathogenesis through several mechanisms, including immune modulation by microbial metabolites and local inflammation initiated by bacterial components in synovial tissue (51).

### 3.1 Gut microbiome dysbiosis regulates inflammatory responses and influences rheumatoid arthritis

In healthy individuals, the gut microbiota is characterized by high diversity and stability, creating a dynamically balanced ecosystem crucial for maintaining gut homeostasis and immune balance (46). Increasing research focuses on the interaction of the gut microbiome in RA patients. The gut microbiota of RA patients undergoes significant alterations, characterized by a decrease in beneficial bacteria and an increase in pathogenic species. This dysbiosis is closely associated with immune system imbalance, driving inflammatory responses and exacerbating RA severity. Studies have demonstrated that in RA patients, significant upregulation is observed in bacterial genera such as Klebsiella, *Enterobacteriaceae*, *Eggerthella*, and *Flavobacteriaceae*, while genera like *Clostridium*, *Blautia*, and *Enterococcus* are downregulated (28). The proportion of Firmicutes is notably reduced in the RA gut, whereas Bacteroidetes levels are relatively increased (28). Firmicutes members, including lactobacilli and bifidobacteria, exert anti-inflammatory effects and modulate immune responses. Conversely, some Bacteroidetes produce toxins and metabolites that exacerbate inflammation and RA. In treatment-naive, new-onset RA patients, *Prevotella* is significantly enriched and shows gene-cluster rearrangement; its 27-kDa protein stimulates a T helper 1 response in 42% of these patients (52, 53). *Lactobacillus* casei has been shown to significantly reduce the expression of interferon-gamma, tumor necrosis factor-alpha, and interleukin-1 beta, thereby preventing joint damage (54). Analysis of stool metabolites in RA patients reveals higher levels of glycerophospholipids, benzene and its derivatives, and cholesterol, while sphingolipids and tryptophan downstream metabolites are found at lower levels (28). In a collagen-induced arthritis mouse model, etanercept significantly decreased the abundance of *Escherichia*/*Shigella* while increasing the relative proportions of *Tannerella*, *Lactobacillus*, and *Clostridium* XIVa. Furthermore, certain natural compounds, such as clematichinenoside, have shown promise in improving RA-related gut dysbiosis and have been proven effective in reducing arthritis symptoms (55).

### 3.2 Impaired intestinal barrier and autoimmune activation

The intestinal barrier, consisting of epithelial cells, tight junction proteins, and mucus layers, primarily functions to prevent pathogens and harmful substances from entering the bloodstream (56, 57). This multilayered barrier is crucial for maintaining gut homeostasis and systemic immune responses. The integrity of the gut barrier is essential for maintaining health. LPS is widely used as a biomarker to assess gut barrier dysfunction, directly indicating the translocation of endotoxins into the bloodstream. Other markers, such as D-lactate and L-lactate, produced by gut bacteria, indirectly reflect changes in gut permeability (58). Barrier proteins, such as zonulin and adhesion molecules, are crucial for maintaining tight junctions, and their circulating levels reflect the functional status of the barrier. When the gut mucosal barrier is impaired, the disruption of tight junctions increases permeability, allowing undigested food particles, toxins, and pathogens to cross the compromised barrier and enter the bloodstream. Impairment of the intestinal barrier leads to tight junction breakdown, increased intestinal permeability, and the translocation of undigested food particles, toxins, and pathogens into the bloodstream, a condition known as leaky gut syndrome (LGS) (59). When these substances enter the bloodstream, they are identified by the immune system as “non-self,” triggering an immune response that produces autoantibodies and inflammatory mediators. LGS initiates inflammatory responses in both the gut and peripheral tissues (60, 61) and is associated with autoimmune disorders such as type 1 diabetes mellitus, multiple sclerosis, rheumatoid arthritis, and celiac disease (62–64). LGS can induce the release of pro-inflammatory cytokines, including tumor necrosis factor-alpha, Interleukin-1β, and Interleukin-6, by activating dendritic and goblet cells. These cytokines can elicit local inflammation and affect the immune system systemically, leading to widespread inflammation and perpetuation (38). Additionally, LGS can enhance autoantigen exposure, stimulate autoimmune responses, and produce autoantibodies, thereby triggering and exacerbating RA.

### 3.3 Immune regulation and inflammatory mediators

The innate immune cells present in the gut-associated lymphoid tissue serve as the primary line of defense against foreign substances in the gastrointestinal tract. Dysbiosis of the gut microbiota can lead to inappropriate activation of these innate immune cells, resulting in an increased production of pro-inflammatory cytokines such as interleukin-12, interleukin-23, and type I interferon, coupled with a concomitant reduction in anti-inflammatory cytokines like transforming growth factor-β and interleukin-10 produced by gut-resident leukocytes (39). In the context of autoimmunity, adaptive lymphocytes, particularly T and B cells, play a pivotal role, with their aberrant activation contributing to the development of RA. Pro-inflammatory antigens originating from the gut can trigger maladaptive responses in the immune system by altering the immune milieu through excessive activation of innate immunity (19). Microbial antigens presented by dendritic cells and goblet cells can activate CD4+ T cells, leading to the differentiation of inflammatory T cell subsets. Notably, Th17 cells, a pro-inflammatory subset of CD4+ T cells, are characterized by their production of interleukin-17 (65). Conversely, CD4+ T cells can also differentiate into Tregs, which aid in suppressing Th17 responses (66, 67). An increased Th17/Treg ratio has been strongly implicated in RA, with this balance being closely regulated by the gut microbiota and its metabolites (39, 68). Furthermore, microbial antigens can stimulate follicular helper T cells, promoting the activation of B lymphocytes and subsequent production of pathogenic autoantibodies that may contribute to the pathogenesis of RA (39). Gut microbiota dysbiosis can trigger the migration of autoreactive cells to the joints, leading to local joint inflammation (69). These autoreactive cells activate macrophages, promoting the production of inflammatory cytokines. Additionally, cytokines such as tumor necrosis factor-α, Interleukin-6, and Interleukin-1 induce fibroblasts to secrete matrix metalloproteinases and receptor activator of nuclear factor κB ligand, further mediating bone and cartilage destruction and driving the progression of RA (70). These intricate interactions underscore the significant role of the gut microbiota in modulating systemic inflammatory responses and suggest that gut dysbiosis, inflammatory cytokines, and immune responses are interconnected factors influencing the development of RA (40).

## 4 Role of the gut-immune system axis in the pathogenesis of rheumatoid arthritis

**Figure 3:**
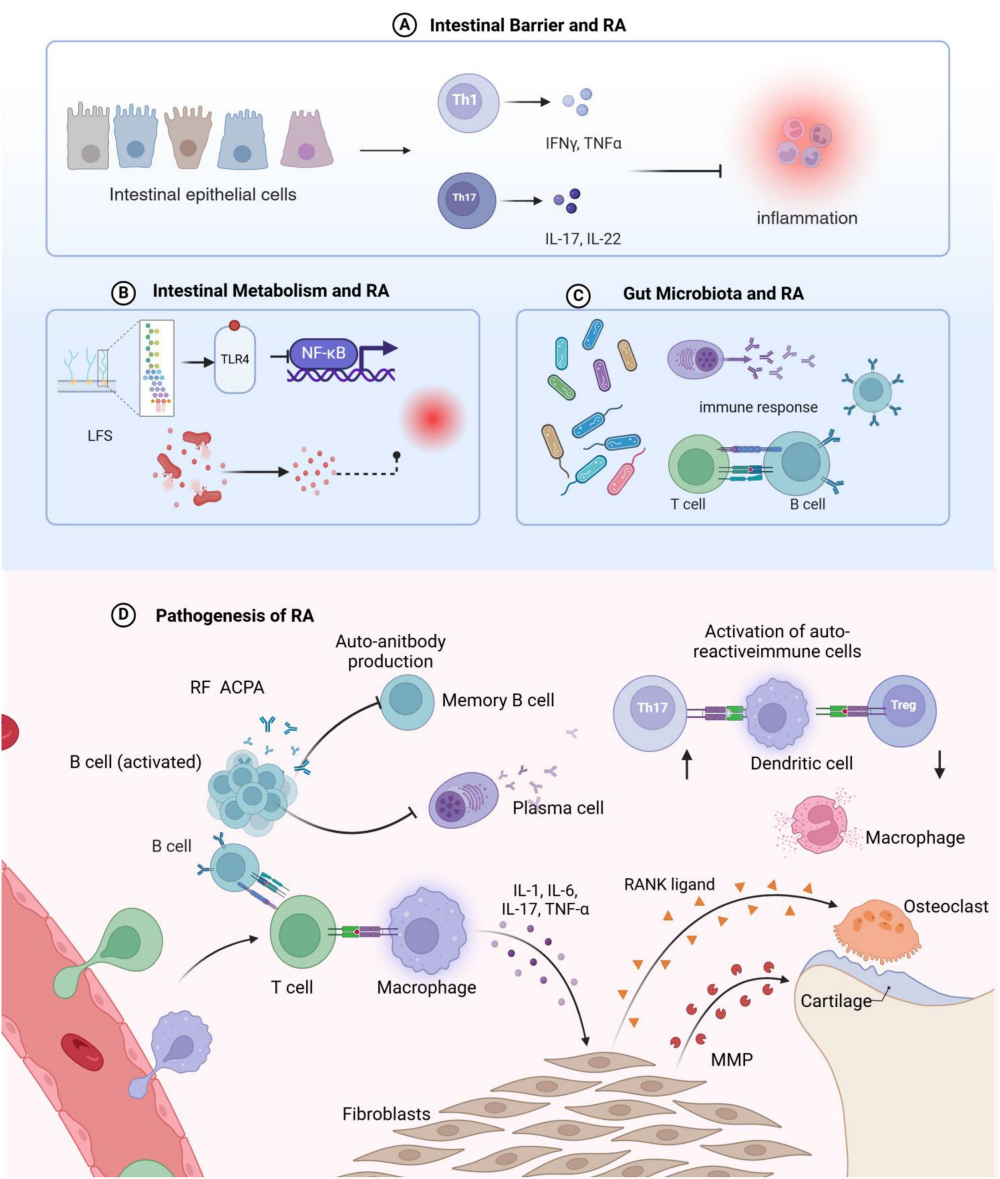
Schematic representation of the mechanisms by which intestinal homeostasis regulates rheumatoid arthritis pathogenesis. **(A)** Disruption of the intestinal barrier leads to increased intestinal permeability, induces T cell-mediated inflammatory responses, and facilitates the migration of autoreactive T cells from the gut to the joints, triggering rheumatoid arthritis in the articular tissues. **(B)** Metabolites derived from the intestinal microbiota can promote systemic inflammation; lipopolysaccharides from intestinal bacteria translocate into the circulation, are recognized by Toll-like receptors, and stimulate the Nuclear Factor kappa-light-chain-enhancer of activated B cells signaling pathway, thereby exacerbating inflammatory processes and contributing to rheumatoid arthritis development. **(C)** Dysbiosis of the intestinal microbiome can perturb the host immune system and its functions, interfere with T cell differentiation, and dysregulate host immune responses. **(D)** Rheumatoid arthritis arises from the convergence of multiple inflammatory pathways, ultimately leading to an imbalanced immune system and perpetuating the chronic inflammatory state.

RA may be intricately linked to how disruptions in gut homeostasis influence immune function through metabolites produced by the gut microbiota (28, 71). Immune T and B cells in the intestinal mucosa have specific phenotypes and functions that are modulated by the microbiota (72). For instance, bacterial peptidoglycan components found in the synovial tissue of RA patients may contribute to inflammation within the joint microenvironment (53, 73). Recent research indicates that changes in gut microbiota composition in RA patients are a crucial factor in initiating abnormal systemic immune responses (38, 74, 75). The gut-immune system axis in the pathogenesis of rheumatoid arthritis shown in **Figure 3**.

### 4.1 Gut microbiota - immune system - rheumatoid arthritis

The mechanisms underlying the association between gut microbiome disturbances and RA have been extensively studied. Imbalance in the intestinal microbiota can impact the host immune system and its functions. The gut microbiota can activate antigen-presenting cells, such as dendritic cells, which alters cytokine production and antigen-presenting processes, interferes with T cell differentiation and function, and modulates the host immune response (76). Gut microbes interact with pattern recognition receptors, key innate immune receptors that detect pathogen-associated molecular patterns, including Toll-like receptors (50). Additionally, gut microbes can promote peptide citrullination through the enzymatic action of peptidyl-arginine deiminase (PAD). The human intestinal epithelium is a primary source of citrullinated peptides, and PAD activity is present in the human gut, with some gut microbes encoding functional microbial PADs (77). Consequently, the intestine can act as a source of citrullinated peptides and other mucosal surface antigens. Citrullination of peptides mediated by *Porphyromonas gingivalis*-expressed PAD is a significant factor in linking periodontitis with increased susceptibility to RA (50, 78). For instance, *Prevotella*-derived Pc-p27 can induce a Th1-mediated immune response by binding to human leukocyte antigen DR molecules in RA patients (2). This association is further supported by immunoglobulin A antibody responses to Pc-p27 in patients with acute and chronic RA, which are linked to the production of Th17 cell factors and anti-citrullinated protein antibodies (53). Moreover, gut microbes influence the host immune system by regulating T cell differentiation and disrupting the balance between Th17 and Treg cells. In a mouse model of RA, specific changes in the gut microbiota may enhance the pathogenic role of Th17 cells while diminishing the inhibitory effects of Tregs thereby promoting Th17-mediated mucosal inflammation (50).

### 4.2 Gut metabolites - immune system - rheumatoid arthritis

When Gram-negative bacteria overgrow, they produce excessive amounts of LPS. Dysfunctional gut microbiota can disrupt gut barrier function, allowing pro-inflammatory substances like LPS to enter the circulatory system through the compromised barrier, thereby triggering systemic inflammation (79). Elevated LPS is recognized by Toll-like receptor 4, which activates the Nuclear Factor kappa-light-chain-enhancer of activated B cells signaling pathway and initiates inflammation (80). Additionally, LPS can activate the complement alternative pathway, which plays a critical role in the development of arthritis, potentially contributing to its progression (81). *Bacteroides* fragilis stimulates T helper 1 responses during early colonization by producing polysaccharide A. It may also contribute to RA pathogenesis by increasing intestinal permeability, reducing tight junction (TJ) protein expression, and affecting epithelial production of interleukin-17A (50, 82). Research shows that SCFAs, such as butyrate, can cross the gut-blood barrier and enter systemic circulation, regulating the function of distant joints and immune cells. In RA patients, SCFA production is reduced, leading to impaired Treg cell function. Lower butyrate levels in the bloodstream are associated with increased inflammatory markers, such as C-reactive protein and interleukin-6, which enhance pro-inflammatory responses and accelerate RA progression. This underscores the direct influence of gut metabolite balance on inflammation. Additionally, fecal analyses reveal a significant reduction in butyrate-producing microbiota in RA patients, further supporting the link between gut microbial metabolism and RA progression (83, 84). Thus, imbalances in gut-derived metabolites directly affect the onset and development of RA through multiple pathways.

### 4.3 Intestinal epithelial barrier - immune system - rheumatoid arthritis

Destruction of the intestinal barrier leads to leakage of intestinal contents, creation of a pro-inflammatory environment, and the activation and infiltration of autoreactive Th17 and other effector T cells (38). Our data indicate that gut barrier dysfunction occurs prior to the clinical onset of arthritis in mouse models (85). Human studies also reveal elevated serum markers associated with impaired gut barrier function before RA onset, which correlates with a higher risk of developing RA later (85). Disruption of the gut barrier allows bacterial components to translocate across the intestinal wall and enter the bloodstream, triggering a robust immune response and inducing the release of pro-inflammatory cytokines, such as tumor necrosis factor-α and interleukin-6 (86). These pro-inflammatory mediators are key drivers of RA pathogenesis, promoting synovial inflammation and tissue destruction.

The TJ between intestinal epithelial cells is crucial for maintaining intestinal barrier integrity. Zonulin, a regulatory protein secreted by intestinal epithelial cells in response to dietary or microbial stimuli, modulates TJ function and intestinal epithelial permeability (87). Zonulin affects the expression of TJ proteins such as Zonula Occludens-1, occludin, and claudin-1 (88). In mouse models, elevated zonulin levels lead to the degradation of essential TJ proteins like Zonula Occludens-1 and an increase in claudin-2 and claudin-15. This combination disrupts TJ integrity, causing significant barrier dysfunction and increased intestinal permeability (89). This disruption not only initiates T-cell-mediated intestinal mucosal inflammation but also facilitates the migration of autoreactive T cells, including T-helper 1 Cells and Th17 cells, from the gut to the joints, thereby triggering RA.

## 5 A novel perspective on rheumatoid arthritis treatment targeting the gut-immune axis

Changes in gut microbiota composition and diversity can affect the onset and progression of RA by influencing the immune system (85). Interventions targeting the gut microbiome in RA patients aim to reshape microbiota composition and diversity, thereby alleviating autoimmune inflammatory responses. Recent studies in rat models have explored the efficacy and safety of strategies like probiotic supplementation and gut barrier stabilization in the management of RA (90).

### 5.1 Development of biomarkers and diagnostic tools - prevention before disease onset

The development of biomarkers and diagnostic tools is essential for the clinical study of RA. Gut microbiota composition and serum cytokine levels are key biomarkers for early diagnosis, monitoring disease activity, and evaluating treatment responses. The gut microbiota in RA patients differs significantly from that in healthy individuals, and these differences could serve as potential markers for early diagnosis (91). Notably, a significant increase in the proportion of Bacteroidetes and a decrease in Firmicutes are characteristic changes in RA (92). Early diagnosis of RA is critical for slowing joint damage. In early-stage RA patients, *Prevotella* abundance increases, while *Bacteroides* decreases (52). Transplanting gut microbiota from early RA patients into germ-free sakuragi mouse model mice induces severe arthritis (93). During active RA, Haemophilus decreases, whereas *Lactobacillus* salivarius increases (94). Furthermore, the relative abundance of *Collinsella* and *Akkermansia* is higher in patients with active RA compared to those in remission (95). These findings suggest that gut microbiota alterations significantly influence RA severity, and shifts in microbial composition could serve as important biomarkers for RA diagnosis. Additionally, variations in serum levels of pro- and anti-inflammatory cytokines can reflect disease activity and treatment efficacy. For instance, elevated levels of tumor necrosis factor-alpha and Interleukin-6 are often associated with increased disease activity, while reduced levels of Interleukin-10 indicate weakened anti-inflammatory mechanisms (19, 96). Comprehensive analysis of gut microbiota and serum cytokine changes can lead to the development of novel diagnostic tools based on the gut-immune axis, supporting early diagnosis and personalized treatment of RA. Recent advancements, such as high-throughput sequencing and metabolomics, enable precise analysis of gut microbiota composition and its metabolites, offering in-depth information on biomarkers. Integrating multi-omics data with machine learning algorithms can enhance diagnostic accuracy and sensitivity. The adoption of these advanced technologies will advance diagnostic tools for RA and strengthen clinical practice.

### 5.2 Treatment of rheumatoid arthritis targeting intestinal homeostasis

RA is a chronic, multisystem autoimmune disease characterized by inflammation in peripheral joints. The primary goals of RA treatment are to alleviate pain and swelling and to control disease progression. Methotrexate is commonly used as the first-line treatment for RA; however, individual sensitivity and tolerance to methotrexate can vary widely. Additionally, methotrexate may accumulate in the kidneys, liver, and pleural and peritoneal effusions, with significant variability in its clearance rate. The human gut microbiota and its enzymatic products influence the bioavailability, clinical efficacy, and toxicity of many drugs through both direct and indirect mechanisms. Moreover, various drugs and active compounds achieve therapeutic effects by normalizing gut microbiota composition, thereby regulating immune cell function (19). An emerging therapeutic approach involves targeting intestinal homeostasis to manage RA, and preliminary clinical studies have shown promising results. Regulating the intestinal microbiota and improving the balance of intestinal homeostasis and immune function offer potential therapeutic benefits for RA.

#### 5.2.1 Microbiome modulators

Probiotics directly modulate the immune system by downregulating Toll-like Receptor expression, thereby reducing inflammation (55). They also regulate antigen-presenting cells. Kwon et al. further demonstrated that probiotics induce a Treg immune response in experimental RA models, significantly promoting the differentiation of T cells into forkhead box P3-expressing Tregs, which play a critical role in regulating and suppressing the inflammatory cascade (97). Additionally, probiotics produce gut metabolites, such as short-chain fatty acids, which have antibacterial and anti-inflammatory properties and can alleviate some symptoms of rheumatoid arthritis (55). Probiotic strains such as *Lactobacillus* and *Bifidobacterium* are known to produce beneficial short-chain fatty acids and contribute to maintaining intestinal mucosal homeostasis (98). Hatakka et al. evaluated the impact of *Lactobacillus* rhamnosus supplementation in patients with stable RA who were not receiving disease-modifying antirheumatic drugs. Although no significant differences were observed in inflammatory markers or clinical disease status compared to the placebo group, patients receiving the probiotic supplementation reported subjective improvements in health (99). In a study conducted RA patients underwent an eight-week probiotic regimen comprising *Lactobacillus* acidophilus, *Lactobacillus* casei, and *Bifidobacterium* bifidum. Compared to the control group, this intervention group exhibited enhanced disease activity scores 28 and reduced levels of high-sensitivity C-reactive protein, suggesting a favorable impact on clinical disease markers (100, 101). Furthermore, a compound probiotic capsule containing *Lactobacillus* casei LC-11, *Lactobacillus* acidophilus LA-14, Lactococcus lactis LL-23, *Bifidobacterium* lactis BL-04, and *Bifidobacterium* bifidum BB-06 also demonstrated therapeutic potential in patients with active RA (102).

Commensal bacteria play a pivotal role in regulating epithelial barrier function. While dysregulated LPS induction can trImmunoglobulin Ger inflammatory responses, microbial byproducts such as butyrate and short-chain fatty acids contribute to maintaining TJ integrity (38). Given the involvement of commensal microbiota in the development of LPS-related autoimmune diseases, interventions targeting the microbiota are emerging as novel strategies for preventing or treating such conditions (103, 104). Secondary bile acids, notably lithocholic acid, have exhibited anti-inflammatory effects in collagen-induced arthritis models, indicating their potential therapeutic role in RA (105). Additionally, propionate derived from *Bacteroides* fragilis may offer an alternative or complementary approach to current RA therapies (106). The dietary indole derivative indole-3-carbinol has been demonstrated to mitigate adjuvant-induced arthritis (107). Sinomenine, a compound that enhances specific gut microbiota-derived indole tryptophan metabolites, activates the aryl hydrocarbon receptor aryl hydrocarbon receptors, and modulates the Nuclear Factor kappa-light-chain-enhancer of activated B cells and mitogen-activated protein kinase pathways, can alleviate RA-associated inflammation and immune responses (108).

#### 5.2.2 Maintenance of intestinal barrier integrity

The zonulin family of peptides is a key regulator of intestinal tight junctions, highly expressed in autoimmune-prone mice and humans, and can predict the transition from autoimmunity to inflammatory arthritis. Elevated serum zonulin levels are strongly associated with gut barrier leakage, malnutrition, and inflammation. Studies suggest that restoring gut barrier function in the pre-arthritis phase using butyrate or type 1 cannabinoid receptor agonists can effectively inhibit the progression of arthritis (85). Treatments aimed at improving intestinal barrier function can help alleviate arthritis symptoms. For instance, microbial metabolites like butyrate are known to regulate epithelial tight junction protein expression effectively and restore intestinal barrier function, highlighting their role in mitigating the impact of microbial dysregulation on barrier integrity (109). Butyrate treatment has been shown to not only restore epithelial barrier function and gastrointestinal permeability to fluorescein Isothiocyanate-dextran but also significantly reduce the incidence of arthritis (109). In preclinical arthritis models, specific inhibition of the zonulin peptide by the antagonist larazotide acetate decreased the development of arthritis by nearly 50% and reduced intestinal permeability, thus preventing immune cell migration from the gut to the joints (85). Therapeutic interventions targeting zonulin can restore tight junction structure, improve barrier function, and partially protect joints from inflammatory damage (110). Early intervention in autoimmune diseases such as rheumatoid arthritis can improve long-term outcomes and increase remission rates. Thus, targeting zonulin and intestinal barrier function early in the disease provides a novel therapeutic strategy for modulating autoimmune development (79). The expression of hypoxia-inducible factor-1αin intestinal epithelial cells is crucial for maintaining barrier integrity. By inhibiting necroptosis in intestinal epithelial cells, hypoxia-inducible factor-1αhelps preserve the intestinal epithelial barrier and is identified as a key regulatory factor in RA treatment (111). There is a potential mechanistic link between disrupted intestinal barrier function and arthritic autoimmune reactions (69, 85, 112, 113). Therefore, reinforcing or restoring intestinal barrier integrity is considered a promising strategy for RA treatment.

## 6 Conclusions

RA is a complex autoimmune disease involving multiple factors, with the gut homeostasis-immune system axis being particularly crucial. The bidirectional regulation between gut homeostasis and the immune system forms a regulatory network that significantly affects RA. RA is closely associated with gut dysbiosis, characterized by an increase in harmful bacteria and a decrease in beneficial bacteria. Gut homeostasis interacts with the immune system to influence RA progression. The gut microbiota and its metabolites regulate immune cells through multiple pathways, affecting immune responses. Gut microbes participate in antigen activation and regulate tight junction proteins to modulate gut mucosal permeability, thereby influencing RA development. Excessive production of metabolites like LPS can damage the gut barrier, promoting inflammation and accelerating RA progression. SCFAs and beneficial bacteria play essential roles in maintaining gut homeostasis and exert anti-inflammatory effects, but their levels are reduced in RA patients. Gut microbiota composition analysis has become a valuable tool for predicting RA susceptibility and controlling disease progression. By integrating gut microbiota data with serum cytokine profiles, new diagnostic approaches based on the gut-immune axis can be developed, offering crucial support for early diagnosis and personalized treatment of RA. Probiotics can induce Treg immune responses, promoting the differentiation of T cells into forkhead box P3-expressing Tregs. Moreover, SCFAs and other metabolites possess antibacterial and anti-inflammatory properties, helping to alleviate RA symptoms.

Therapeutic strategies targeting the gut microbiome have shown potential in modulating the immune system, offering new avenues for RA treatment. However, several limitations remain: the exact mechanisms by which specific microbiota or their metabolites influence RA pathogenesis through immune modulation are not fully understood; the molecular processes linking dysbiosis or intestinal barrier disruption to systemic inflammation and RA need further elucidation. The impact of intestinal homeostasis on arthritis and its underlying mechanisms requires more detailed investigation. The clinical efficacy and safety of RA treatments that focus on regulating intestinal homeostasis need to be thoroughly evaluated and validated. Future research should include additional clinical studies to confirm the effects and mechanisms of gut microbiota regulation in RA prevention and treatment. Investigating the action mechanisms of specific probiotics or metabolites, developing novel microbiome-targeted therapies, and integrating traditional drug treatments with lifestyle interventions could enhance the comprehensive management of RA. Further exploration of drug metabolism in RA and the interplay between gut homeostasis and the immune system is also needed. Advancing our understanding of strategies to regulate intestinal homeostasis may offer new insights and therapeutic targets, improving patient outcomes and quality of life.
